# A computational study on outliers in world music

**DOI:** 10.1371/journal.pone.0189399

**Published:** 2017-12-18

**Authors:** Maria Panteli, Emmanouil Benetos, Simon Dixon

**Affiliations:** Centre for Digital Music, School of Electronic Engineering and Computer Science, Queen Mary University of London, London, United Kingdom; University of Connecticut, UNITED STATES

## Abstract

The comparative analysis of world music cultures has been the focus of several ethnomusicological studies in the last century. With the advances of Music Information Retrieval and the increased accessibility of sound archives, large-scale analysis of world music with computational tools is today feasible. We investigate music similarity in a corpus of 8200 recordings of folk and traditional music from 137 countries around the world. In particular, we aim to identify music recordings that are most distinct compared to the rest of our corpus. We refer to these recordings as ‘outliers’. We use signal processing tools to extract music information from audio recordings, data mining to quantify similarity and detect outliers, and spatial statistics to account for geographical correlation. Our findings suggest that Botswana is the country with the most distinct recordings in the corpus and China is the country with the most distinct recordings when considering spatial correlation. Our analysis includes a comparison of musical attributes and styles that contribute to the ‘uniqueness’ of the music of each country.

## Introduction

With the increasing accessibility of large sound archives and advances in Music Information Retrieval (MIR) technologies [1] it is possible to automatically analyse vast amounts of sound recordings. This has been the target of several MIR studies, usually with a two-fold scope: first, the development of technology for the analysis of music audio, and second, the application of technology to study musical phenomena. While the development of MIR technologies has been advancing, few studies have attempted to apply it to the analysis of large corpora of folk and traditional music. We are interested in a large-scale comparison of world music with particular focus on music similarity and distinctiveness.

In the field of ethnomusicology, several studies have considered the comparison of world music cultures [2, 3]. Data collection and annotation for this type of research is usually done manually by ethnomusicologists, a process which limits the potential for large-scale results. In the field of MIR, large-scale comparative studies have focused mainly on Eurogenetic music [4, 5], where Eurogenetic defines music styles of mainly Western traditions for example classical and popular repertoires. The study of non-Eurogenetic music using computational tools falls under the emerging field of Computational Ethnomusicology [6, 7]. While several research projects have focused on the development of MIR tools for world music analysis [8–12], no study, to the best of our knowledge, has applied such computational methods in the analysis of a large world music corpus.

Music similarity lies at the heart of most MIR applications, such as music classification, retrieval and recommendation [1]. In this study, we focus on music dissimilarity or musical distinctiveness. In particular we aim to detect music outliers. Outlier detection is a common pre-processing step in the analysis of big data collections [13]. In music, outlier detection can reveal recordings with outstanding musical characteristics. Tracing the geographic origin of these recordings could help identify areas of the world that have developed a unique musical character. Due to the long-lasting traditions of orally-transmitted repertoires and the lack of scores or consistent notation in world music, our music data is extracted solely from the audio. Music similarity/dissimilarity in this case is modelled by considering musical attributes captured in the audio signal.

In previous work we have explored the suitability of audio features for music similarity and content description [14]. Audio features for the purpose of studying world music need to be agnostic to style characteristics so that they can generalise to the diversity of music styles. We found rhythmic and melodic descriptors that are invariant to tempo and pitch transformations and are fairly robust to transformations of the recording quality. We used these features in combination with feature learning to assess music similarity in a relatively small world music corpus [15] as well as to detect and analyse music outliers in a preliminary study [16].

In this study we expand prior work to world music analysis using a larger corpus and evaluating additional methods. We use signal processing tools to process audio data from a collection of recorded world music. Machine learning and data embeddings are used to learn a feature space of music similarity. Data mining techniques are applied to detect outliers in this space. Results are evaluated quantitatively using metrics to assess classification accuracy and qualitatively via visualisation of the space and listening to audio examples. Our observations on music similarity comply with expected geographical and cultural links whereas outliers provide insights on the evolution of world music. This is the first study to investigate outliers in world music with such a large scale. Our developments contribute to defining concepts and methods from which future work in the study of large world music corpora can benefit.

This paper is organised as follows. The Related work section provides a literature review of related studies and methods. The Methodology section describes the materials and tools used in this study. It focuses on details of the music corpus under investigation, audio feature extraction and feature learning methods for music similarity, and data mining techniques to assess music similarity and distinctiveness as well as methods for modelling spatial relations. Results are presented in the Results section and limitations of the study as well as directions for future improvement are considered in the Discussion section. Findings are summarised in the Conclusion section.

## Related work

### Comparison of world music cultures

The comparison of world music cultures has been the topic of several ethnomusicological studies since the beginning of the 20th century [2, 3, 17, 18]. Alan Lomax, one of the major comparativists, made more than 4000 recordings from around the world and annotated their performance-style characteristics based on the system of ‘Cantometrics’ [2, 17]. Using a phylogenetic analysis, he formed the hypothesis that there are two music evolutionary roots, the eastern Asian and the Sub-Saharan African music cultures from which all other music styles have possibly evolved [17]. In a similar manner, Savage et al. [3] analyse 304 recordings from the Garland Encyclopedia of Music [19] using the annotation system of ‘Cantocore’ [20] in addition to the Cantometrics descriptors. In this study, Savage et al. show that there are no ‘absolute’ music universals, i.e., music properties that are shared amongst all music of the world without exceptions, but rather ‘statistical’ universals, i.e., properties that occur with exceptions but are statistically consistent in music from around the world. This supports the hypothesis of the current study, that there are outliers, pieces outside the statistical norms shared by much of the world’s music.

Applications of comparative musicology have also focused on contrasting music styles to genetic and language evolution [3, 18, 21–23]. The study of 220 traditional songs from 9 indigenous populations from Taiwan [18] showed that population structure for genetics exhibits stronger parallels to music than to language. The study of 700 recordings from 58 patrimonies of rural areas in Gabon [23] found that there is a predominant vertical transmission of musical characteristics such as metre, rhythm, and melody, where vertical transmission refers to the inheritance from ancestors in contrast to the horizontal exchange between neighbours.

### Large-scale music corpus analysis

Computational approaches to music analysis enable the study of larger music corpora. Large-scale MIR studies have focused on the analysis of popular (mainly Eurogenetic) music [4, 5, 24]. For example, Serra et al. [4] analysed pitch, loudness and timbre characteristics in 464411 recordings of contemporary Western popular music between 1955−2010 and found that over the years music shows less variety in pitch transitions, consistent homogenisation of the timbral palette, and louder and potentially poorer volume dynamics. A related study of 24941 Western popular music recordings between 1922−2010 showed that the most influential songs were more innovative during the early 1970s and the mid 1990s [24]. Mauch et al. [5] analysed 17094 songs from the US Billboard Hot 100 between 1960−2010 and found that pop music evolved with particular rapidity during three stylistic ‘revolutions’; around 1964, 1983 and 1991. Other corpus analysis studies have focused on the automatic classification of music by genre [25–27] via the combination of different audio features.

Fewer studies have considered the computational analysis of non-Western music corpora [12, 28]. Moelants et al. [12] analysed pitch distributions of 901 recordings from Central Africa and found that recent recordings exhibit Western-influenced scales. Gómez et al. [28] studied aspects of timbre, rhythm, and tonality in 5905 recordings from Western and non-Western music styles and showed that Western music is more equal-tempered than non-Western music. A comparison between music features and geographical latitude and longitude showed that latitude is mostly associated with tonal features whereas longitude with rhythmic ones. A number of studies have considered automatic classification of non-Western music styles. Liu et al. [29] classify 1300 music recordings into six cultural styles using timbre, rhythm, wavelet coefficients and musicology-based features. Kruspe et al. [30] study the automatic classification of 4400 recordings from non-Western music traditions into 9 geographical areas using features of timbre, rhythm and tonality. Zhou et al. [31] use a corpus of 1142 non-Western music tracks from 73 countries and predict the geographical location of each track via a regression method.

### Computational approaches to music similarity

Music similarity is studied in several MIR application areas including automatic genre classification [32], cover song detection [33], structural segmentation [34], pattern recognition [35] and music recommendation [36]. In the Music Information Retrieval Evaluation eXchange (MIREX), the annual public evaluation of MIR systems and algorithms, there is a task on Audio Music Similarity [37]. Since music is a multifaceted concept the study of music similarity is often divided into separate aspects [38]. For example, studies have focused on developing tools and datasets to investigate similarity in aspects of melody [39–41], rhythm [42–44], timbre [45–47], or harmony [48, 49].

The assessment of music similarity is subjective. Automatic systems built for music similarity tasks often need to be trained on a ground truth obtained from human listeners. Several approaches have used genre labels as a proxy for similarity [27]. In this case the assumption is made that songs from the same genre exhibit similar music characteristics. Other studies have focused on the creation of a ground truth set via the collection of similarity ratings from human listeners [50]. Given the scarcity of ground truth data, the evaluation of music similarity systems and the suitability to generalise to all music has been challenged [51, 52]. For example, music similarity systems that are evaluated based on the classification accuracy of genre labels are demonstrated to learn irrelevant music attributes [51]. On the other hand, music similarity systems evaluated with judgements from human listeners are limited by the inter-rater agreement [52]. In particular, due to the challenges in the definition of music similarity and the subjectivity of the task there is often a low inter-rater agreement. As computational models are not expected to outperform the level of human agreement there exists an upper bound beyond which the performance of the model cannot be further improved. Therefore the development and evaluation of a music similarity system still remains a challenge, especially in the yet unexplored space of world music.

### Outliers in big data collections

Outlier detection is an essential step in the analysis of big data collections [53]. Outliers denote data points that deviate significantly from the distribution and often need to be filtered out or treated in a different manner. Applications of outlier detection include, amongst others, the identification of intrusions in computer networks [54], fraud in credit cards [55] and abnormal symptoms in disease diagnosis [56]. The study of outliers with respect to spatial relations, as assumed in this music research, adopts concepts of spatial statistics. A spatial outlier is usually viewed as a local anomaly whose non-spatial attribute values are extreme compared to its neighbours [57]. Spatial outlier detection can help locate extreme meteorological events [58], identify disease outbreaks [59], and predict crime hot spot areas [60].

The detection of outliers in music data is still a new area of research. Bountouridis et al. [61] investigate outlier detection in music data using multiple sequence alignment techniques. Lu et al. [62] compare outlier detection techniques applied on a music genre recognition dataset. Hansen et al. [63] apply outlier detection using probability density estimation methods to clean up large-scale datasets of mislabelled data. Livshin and Rodet [64] use outlier detection methods to identify badly recorded musical instrument samples. In the current study, outlier detection is used to identify geographical regions with distinct musical characteristics.

## Methodology

**Fig 1:**
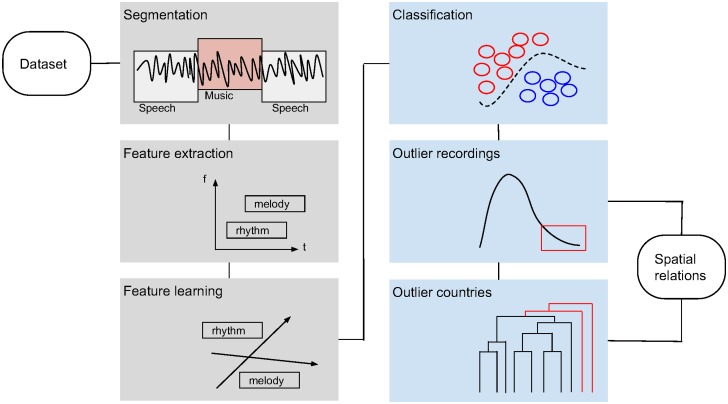
Overview of the methodology.

The methodology is summarised as follows. For each audio recording in our dataset we extract music descriptors by a) filtering out speech segments as detected via a speech/music discriminator algorithm, b) extracting audio descriptors capturing aspects of music style, c) applying feature learning to reduce dimensionality and project the recordings into a similarity space. We optimise parameters and evaluate music similarity in the projected space by a classification task. The projected space is used to identify recordings that are outliers. We refer as ‘outliers’ to the recordings that stand out with respect to the whole set of recordings. Outliers are detected for different sets of features focusing on rhythm, melody, timbre, or harmony and a combination of these. We take into account spatial relations to form geographical neighbourhoods and use these to detect spatial outliers, i.e., recordings that stand out with respect to their neighbours. Lastly, we extract a feature representation for each country by summarising information of its recordings. Hierarchical clustering is used to get an overview of similarity and dissimilarity between countries. The methodology is summarised in Fig 1 and explained in detail in the sections below.

In our analyses we use the country label of a recording as a proxy for music style. We assume that recordings originating from the same country have common musical characteristics and we use this as the ground truth to train our models. However, it is often the case that a music style is not unique to a single country. Music styles may be shared across many countries and a country may exhibit several music styles. The reason for choosing country as the unit of analysis in this study is two-fold: First, country label is the most consistent information available in our music metadata compared to, for example, music genre, language, or culture information (see also Data section). Second, several studies have considered larger geographical regions (e.g., continents or cultural areas) for the comparison of music styles [28, 30, 65]. Country boundaries work in a similar way but provide a more fine-grained unit for analysis. Alternative approaches are discussed further in the Discussion section.

### Data

We aim to investigate music similarity in a world music corpus. The notion of world music is ambiguous often mixing folk, popular, and classical musics from around the world and from different eras [66]. In this study world music refers to recorded material from folk and traditional music styles from around the world. In particular we focus on field recordings collected by ethnomusicologists since the beginning of the 20*th* century. Our music dataset is drawn from two large archives, the Smithsonian Folkways Recordings [67] and the World & Traditional music collection from the British Library Sound Archive [68]. Both archives include thousands of music recordings collected over decades of ethnomusicological research.

Even though access to large collections of world music recordings is now feasible, the creation of a representative world music corpus is still challenging. An ideal world music corpus would include samples from all inhabited geographical regions and provide information on the spatio-temporal and cultural origins of each music piece. The samples chosen would have to be sufficient to represent the diversity of styles within each music culture and the corpus as a whole should be a balanced collection of music cultures. Given the archives available today, the challenges in corpus creation involve addressing what defines a good sample, how to balance the diverse styles represented in the collection, how to avoid the Western-music bias and how to maximize the size of the corpus. These challenges have also been the main point of criticism for several music comparative studies [69–72]. Our effort to create a world music corpus from the currently available data is described below.

We use a subset of the Smithsonian Folkways Recordings collection which consists of more than 40000 audio recordings, including music as well as poetry. It has a large representation from North America (more than 21000 from the United States and around 1400 from Canada). It also includes around 7700 recordings from Eurasia (1700 from the United Kingdom, 800 from Russia, 800 from France), 4200 recordings from South America (Mexico 600, Trinidad and Tobago 400, Peru 400), 2300 from Asia (India 400, Indonesia 400, Philippines 200, China 200), 1900 from Africa (South Africa 200, Ghana 200, Kenya 100), and 400 from Oceania. Recording dates span from 1938 to 2014. We also use a subset of the World & Traditional music collection of the British Library Sound Archive as curated for the purposes of the Digital Music Lab project [8]. This subset consists of more than 29000 audio recordings with a large representation (17000) from the United Kingdom. It also includes around 7300 recordings from Africa (mostly from Uganda 3000), 2300 from Asia (mostly from Nepal 800 and Pakistan 700), and less than 1000 recordings from Oceania, North and South America. Recording dates span from 1898 to 2014. The metadata associated with each music recording include the country where the recording was made and the year it was recorded, the language and sometimes cultural background of the performers, the subject of the music or short description of its purpose, the title, album (if any), and information of the collector or collection it was accessed from.

In the above archives there is an unbalanced representation of music cultures, with the majority of recordings originating from Western-colonial areas. What is more, metadata for each recording is not always present or is inconsistent. To create a corpus we sample recordings based on the country information which in this case is more consistent than other culture-related metadata. In order to ensure geographical spread we require recordings from as many countries as possible. We set a minimum requirement of *N*_*min*_ = 10 recordings from each country and select a maximum of *N*_*max*_ = 100. Setting the minimum to 10 recordings is a trade-off between allowing under-represented areas to be included in the dataset and having a sufficient number of samples for each country. Although a sample of 10 recordings is too small to represent the diversity of music styles within a country, raising this minimum to e.g. 50 would exclude many of the countries we currently analyse and would limit the geographical scope of the study. Setting the maximum to 100 recordings prevents the over-represented areas from dominating the corpus. We sample at random *N* recordings from each country, where *N* is bounded by *N*_*min*_ and *N*_*max*_ as explained above.

Since the medium of analysis is digitised audio, most of our samples are dated since the 1950*s*, with the exception of some recordings from the British Library collection dated around 1900 which were digitised from wax cylinders. The duration of audio recordings from the Smithsonian Folkways Recordings collection is restricted to 30 seconds since we use the publicly available 30-second audio previews. For the British Library Sound Archive data we have access to complete recordings but we only sample the first music segments up to a total duration of 30 seconds for consistency with the short audio excerpts of the Smithsonian Folkways collection.

**Fig 2:**
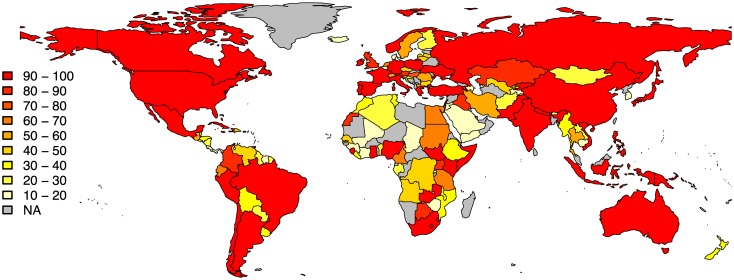
The distribution of countries in our dataset of 8200 world music recordings.

**Fig 3:**
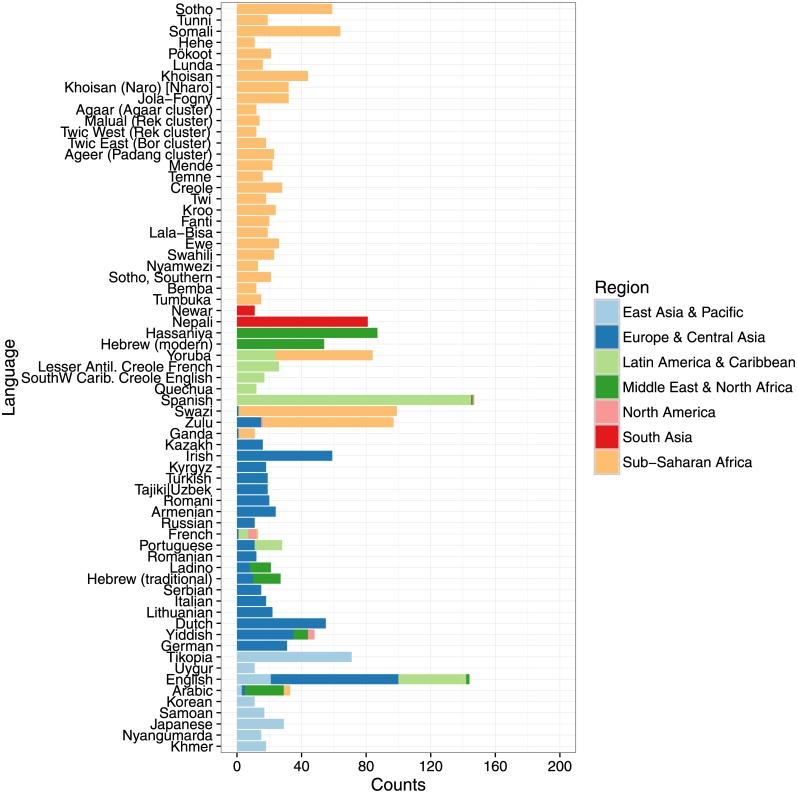
The languages in our world music corpus which are represented by a minimum of 10 recordings.

**Fig 4:**
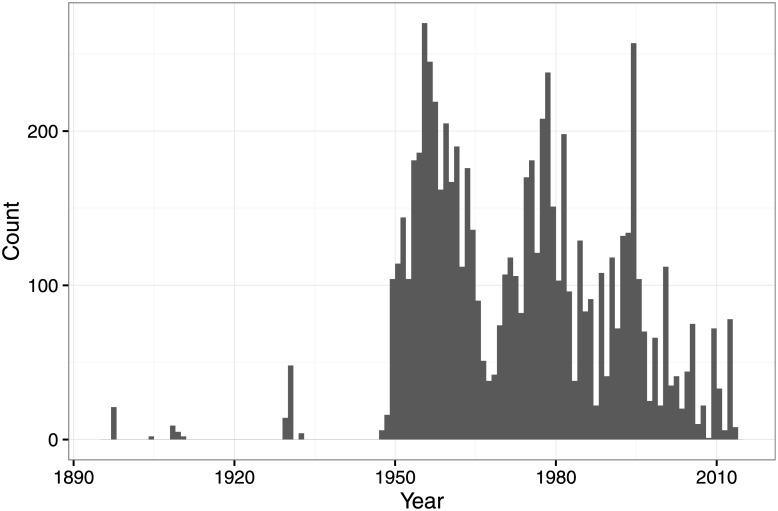
The time span of recordings in our world music corpus.

Given the above criteria, the final collection consists of a total of 8200 recordings, 6132 from the Smithsonian Folkways Recordings collection and 2068 from the British Library Sound Archive collection. The recordings originate from 137 countries with mean 59.9 and standard deviation 33.8 recordings per country (Fig 2). A total of 67 languages is represented by a minimum of 10 recordings, with a mean of 33.5 and standard deviation of 33.5 recordings per language (Fig 3). The recordings span the years between 1898−2014 with median year 1974 and standard deviation of 17.9 years (Fig 4).

### Audio content analysis

Over the years several toolboxes have been developed for music content description [73–76]. Applications of these toolboxes include tasks of automatic classification and retrieval of mainly Eurogenetic music (Related work section). Audio content analysis of world music recordings has additional challenges. First, the audio material is recorded under a variety of recording conditions (live and field recordings), and is preserved to different degrees of fidelity (old and new recording media and equipment). Second, the music is very diverse and music descriptors designed primarily for Eurogenetic music might fail to capture particularities of world music styles. Our audio content analysis process includes a pre-processing step to remove speech segments from the dataset (Pre-processing section) and low-pass filtering to reflect limitations of old recording equipment (Features section). With respect to music descriptors, between specifically designing them as in other comparative music studies [28, 30, 31] and automatically deriving them from the spectrogram [77, 78] we choose a middle ground. We use expert knowledge to derive low-level music representations (Features section) and combine them with feature learning methods (Feature learning section) to adapt the representation to particularities of the music we analyse. Details for each step of the audio content analysis process are provided below.

#### Pre-processing

Our dataset consists of field recordings that sometimes mix speech and music segments. We are only interested in music segments but due to the lack of metadata speech segments cannot be filtered out a-priori. An essential pre-processing step is therefore the discrimination between speech and music segments. By speech/music segmentation we refer to the detection of segment boundaries and the classification of the segment as either speech or music. The task of speech/music segmentation has been the focus of several studies in the literature [79–81] and it was also identified as a challenge in the 2015 Music Information Retrieval Evaluation eXchange (MIREX) [82]. We select the best performing algorithm [83] from the MIREX 2015 evaluation. As part of the MIREX 2015 evaluation, the algorithm was tested on a non-overlapping set of British Library recordings which is very similar to the recording collection we use in this study and achieved a frame-based F-measure of 0.89. The algorithm is based on summary statistics of low-level features including Mel frequency cepstrum coefficients (MFCCs), spectral entropy, tonality, and 4 Hertz modulation, and is trained on folk music recordings [84]. We apply this algorithm to detect speech/music segments for all recordings in our dataset and use solely the music segments of each recording for further analysis. In case of long audio excerpts we only select the initial music segments up to a total duration of maximum 30 seconds (see also duration of recordings in Data section).

#### Features

We are interested in descriptors capturing aspects of world music style. We adopt the notion of music style by Sadie et al. [85], ‘style can be recognized by characteristic uses of form, texture, harmony, melody, and rhythm’. The use of form is ignored in this study as most of our music collection is restricted to short audio excerpts rather than complete recordings. We focus on state of the art descriptors (and adaptations of them) that aim at capturing relevant rhythmic, timbral, melodic, and harmonic content. In particular, we extract onset patterns with the scale transform [86] for rhythm, pitch bi-histograms [87] for melody, average chromagrams [88] for harmony, and Mel frequency cepstrum coefficients (MFCCs) [89] for timbre content description. We choose these descriptors because they define low-level representations of the musical content, i.e., a less detailed representation but one that is more likely to be robust with respect to the diversity of the music styles we consider. In addition, these features achieved state-of-the-art performances in relevant classification and retrieval tasks [14], for example, onset patterns with the scale transform perform best in classifying Western and non-Western rhythms [90, 91] and pitch bi-histograms have been applied successfully in (melody-based) cover song recognition [87].

**Fig 5:**
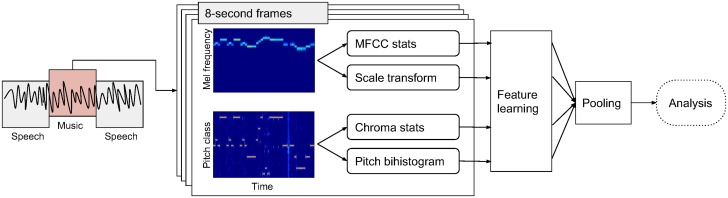
Overview of the audio content analysis process. Mel-spectrograms and chromagrams are processed in overlapping 8-second frames to extract rhythmic, timbral, harmonic, and melodic features. Feature learning is applied to the 8-second features and average pooling across time yields the representations for further analysis.

The audio features used in this study are computed with the following specifications. All recordings in our dataset have a sampling rate of 44100 Hz. For all features we compute the (first) frame decomposition using a window size of 40 ms and hop size of 5 ms. The output of the first frame decomposition is a Mel spectrogram and a chromagram. We use a second frame decomposition to extract descriptors over 8-second windows with 0.5-second hop size. This is particularly useful for rhythmic and melodic descriptors since rhythm and melody are perceived over longer time frames. Rhythmic and melodic descriptors considered in this study are derived from the second frame decomposition with overlapping 8-second windows. Timbral and harmonic descriptors are derived from the first frame decomposition with 0.04-second windows and for consistency with rhythmic and melodic features, they are summarised by their mean and standard deviation over the second frame decomposition with overlapping 8-second windows. The window of the second frame decomposition is hereby termed as ‘texture window’ [25]. The window size *w* of the texture window was set to 8 seconds after the parameter optimisation process described in the Parameter optimisation section. For all features we use a cutoff frequency at 8000 Hz since most of the older recordings do not contain higher frequencies than that. The audio content analysis process is summarised in Fig 5.

**Rhythm and Timbre.** For rhythm and timbre features we compute a Mel spectrogram with 40 Mel bands up to 8000 Hz using Librosa [76]. To describe rhythmic content we extract onset strength envelopes for each Mel band and compute rhythmic periodicities using a second Fourier transform with window size of 8 seconds and hop size of 0.5 seconds. We then apply the Mellin transform to achieve tempo invariance [90] and output rhythmic periodicities up to 960 beats per minute (bpm). The output is averaged across low and high frequency Mel bands with cutoff at 1758 Hz. The resulting rhythmic feature vector has length 400 values. Timbral aspects are characterised by 20 MFCCs and 20 first-order delta coefficients after removing the DC component [89]. We take the mean and standard deviation of these coefficients over 8-second windows with 0.5-second hop size. This results in a total of 80 feature values describing timbral aspects.

**Harmony and Melody.** To describe harmonic content we compute chromagrams using variable-*Q* transforms [92] up to 8000 Hz with 5 ms hop size and 20-cent pitch resolution to allow for microtonality. Chromagrams are aligned to the pitch class of the maximum magnitude per recording for key invariance. Harmonic content is described by the mean and standard deviation of chroma vectors using 8-second windows with 0.5-second hop size. The dimensionality of the harmonic feature vector results in a total of 120 values. To describe melodic content we extract pitch contours from polyphonic music signals using a method based on a time-pitch salience function [93]. The pitch contours are converted to 20-cent resolution binary chroma vectors with entries of 1, whenever a pitch estimate is active at a given time, and 0 otherwise. Melodic aspects are captured via pitch bi-histograms which denote counts of transitions of pitch classes [87]. We use a window of *d* = 0.5 seconds to look for pitch class transitions in the binary chroma vectors. The resulting pitch bi-histogram matrix consists of 3600 = 60 × 60 values corresponding to pitch transitions with 20-cent pitch resolution. For efficient storage and processing, the matrix is decomposed using non-negative matrix factorisation [94]. We keep 2 basis vectors with their corresponding activations to represent melodic content. It was estimated that keeping only 2 bases was enough to provide sufficient reconstruction for most pitch bi-histogram matrices in our dataset (average reconstruction error < 25%). Pitch bi-histograms are also computed over 8-second windows with 0.5-second hop size. This results in a total of 120 feature values describing melodic aspects.

Combining all features together results in a total of 840 descriptors for each recording in our dataset. A *z*-score standardisation of the 840 features is applied across all recordings before further processing.

#### Feature learning

For the low-level descriptors presented in the Features section we aim to learn high-level representations that best characterise music style similarity. Feature learning is also appropriate for reducing dimensionality, an essential step for the amount of data we analyse. We learn feature representations from the 8-second frame-based descriptors. In our analysis we consider the country label of a recording as a proxy for style and use this for supervised training and cross-validating our methods.

There are numerous feature learning techniques to choose from in the literature. Non-linear models such as neural networks usually require large training data sets [95]. We have a fairly limited number of audio recordings and our low-level descriptors partly incorporate expert knowledge of the music (section Features). In this case, simpler feature learning techniques are more suitable for the amount and type of data we have. We explore the applicability of 4 linear models trained in supervised and unsupervised fashions.

The audio features are standardised using *z*-scores and aggregated to a single feature vector for each 8-second frame of a recording. Feature representations are learned using Principal Component Analysis (PCA), Non-Negative Matrix Factorisation (NMF), Semi-Supervised Non-Negative Matrix Factorisation (SSNMF), and Linear Discriminant Analysis (LDA) methods [94]. PCA and NMF are unsupervised methods and try to extract components that account for the most variance in the data without any prior information on the data classes. LDA is a supervised method and tries to identify attributes that account for the most variance between classes (in this case country labels). SSNMF works similarly to NMF with the difference that ground truth labels are taken into account in addition to the data matrix in the optimisation step [96].

We split the 8200 recordings of our collection into training (60%), validation (20%), and testing (20%) sets. We train and test our models on the frame-based descriptors; this results in a dataset of 325435, 106632, and 107083 frames for training, validation, and testing, respectively. Frames used for training do not belong to the same recordings as frames used for testing or validation and vice versa. We use the training set to train the PCA, NMF, SSNMF, and LDA models and the validation set to optimise the parameters. In each experiment we retain components constituting to 99% of the variance. In the Results section we analyse the feature weights for the components of the best performing feature learning method.

A classification task is used to assess the quality of the learned space and optimise the parameters. An ideal music similarity space separates well data points belonging to different music classes and good classification results can be achieved with simple classifiers. We are not interested to build a powerful classifier since our primary aim is to assess the learned embeddings and not to optimise the classification task itself. We therefore focus on classifiers widely used in the machine learning community [97]. We train 4 classifiers, K-Nearest Neighbour (KNN), Linear Discriminant Analysis (LDA), Support Vector Machines (SVM), and Random Forest (RF), to predict the country label of a recording. The purpose of the classification task is to optimise the window size *w* of the audio descriptors and assess the quality of the learned spaces in order to select the optimal feature learning method for our data. We use the classification F-score metric to compare the performance of the models. In the Results section we also analyse the coefficients of the best performing classifier.

In order to assess the contribution of different features to the classification task we consider 5 sets of features: a) scale transform (rhythmic) b) MFCCs (timbral), c) average chroma vectors (harmonic), d) pitch bi-histograms (melodic), and e) the combination of all the above. In each case, feature learning is applied on the selected feature set and frame-based projections are aggregated using the mean prior to classification. We also tested for aggregation using the mean and standard deviation of frame-based descriptors but this did not improve results; hence it was omitted. In the case of testing the combination of all features (e), we first reduce dimensionality for each feature set separately and then concatenate the components from all feature sets before mean aggregation and classification. Results for the feature learning optimisation and classification experiments are presented in the Results section.

### Data mining

#### Outlier recordings

The feature learning and classification methods described above (Feature learning section) identify the optimal projection for the data. In the next step of the analysis we use the projected space to investigate music dissimilarity and identify outliers in the dataset. A recording is considered an outlier if it is distinct compared to the whole set of recordings. We detect outliers based on a method of squared Mahalanobis distances [13, 98]. Using Mahalanobis, a high-dimensional feature vector is expressed as the distance to the mean of the distribution in standard deviation units. Let $X\in R^{I\times J}$ denote the set of observations for *I* recordings and *J* features. The Mahalanobis distance for observation **x**_**i**_ = (*x*_1_, *x*_2_, …, *x*_*J*_)^*T*^ for recording *i* from the set of observations *X* with mean *μ* = (*μ*_1_, *μ*_2_, …, *μ*_*J*_)^*T*^ and covariance matrix *S* is denoted

$$\begin{array}{c} D_{M}\left(x_{i}\right)=\sqrt{{\left(x_{i}-\mu \right)}^{T}S^{-1}\left(x_{i}-\mu \right)}. \end{array}$$ (1)

Data points that lie beyond a threshold, typically set to the *q* = 97.5% quantile of the chi-square distribution with *J* degrees of freedom [99], are considered outliers. This is denoted

$$\begin{array}{c} O=\{i\in H|D_{M}\left(x_{i}\right)>\sqrt{\chi_{J,q}^{2}}\} \end{array}$$ (2)

where *H* = {1, 2, …, *I*} denotes the index of the observations.

Due to the high dimensionality of our feature vectors every data point can be considered far from the centre of the distribution [100]. To compensate for a possible large amount of outliers we consider a higher threshold based on the *q* = 99.9% quantile of the chi-square distribution.

To gain a better understanding of the type of outliers for each country we detect outliers using a) rhythmic, b) timbral, c) harmonic, and d) melodic features. For example, for *J*_*R*_ the dimensionality of the rhythmic feature vector and $X_{R}\in R^{I\times J_{R}}$ the set of observations, the set of outlier recordings with respect to rhythmic characteristics is denoted

$$\begin{array}{c} O_{R}=\left\{i\in H|D_{M}\left(x_{R,i}\right)>\sqrt{\chi_{J_{R},99.9}^{2}}\right\} \end{array}$$ (3)

for observation **x**_*R*,*i*_ ∈ *X*_*R*_. We detect outliers with respect to rhythmic (*O*_*R*_), timbral (*O*_*T*_), melodic (*O*_*M*_), and harmonic (*O*_*H*_) characteristics.

#### Spatial neighbourhoods

In the previous section outliers were detected by comparing a recording to all other recordings in the dataset. Here we take into account spatial relations and compare recordings from a given country only to recordings of its neighbouring countries. In this way we are able to identify spatial outliers, i.e. recordings that are outliers compared to their spatial neighbours [57]. We construct spatial neighbourhoods based on contiguity and distance criteria: a) two countries are neighbours if they share a border (a vertex or an edge of their polygon shape), b) if a country doesn’t border with any other country (e.g., the country is an island) its neighbours are defined by the 3 closest countries estimated via the Euclidean distance between the geographical coordinates (latitude and longitude) of the centre of each country.

Let *N*_*i*_ denote the set of neighbours for country *i* estimated via

$$\begin{array}{c} N_{i}=\left\{j\in \left\{1,\ldots ,R\right\}|\text{j}\;\text{is}\;\text{neighbour}\;\text{to}\;\text{i}\right\} \end{array}$$ (4)

for *R* the number of countries. The spatial neighbourhood is represented as a weight matrix $W\in R^{R\times R}$ where entry *w*_*ij*_ ∈ *W* is non-zero whenever country *j* is neighbour to country *i*. This is denoted

$$\begin{array}{c} w_{ij}=\{\begin{array}{cc} \frac{1}{n_{i}}, & \text{if}\;j\in N_{i}\\ 0, & \text{otherwise} \end{array} \end{array}$$ (5)

where *n*_*i*_ = |*N*_*i*_| denotes the total number of neighbours for country *i*. By definition, weight matrix W is row-standardized, $\sum_{j=1}^{R}w_{ij}=1$.

Table in S1 Table provides the neighbours of each country as estimated via this approach. The geographical boundaries of each country are derived from spatial data available via the Natural Earth platform [101].

The set of recordings from a given country is appended with recordings from neighbouring countries as defined by the country’s spatial neighbourhood (S1 Table). This set is used to detect outliers with the Mahalanobis distance as defined in Eq 2. Spatial outliers are detected in this manner for all countries in our dataset.

#### Outlier countries

The unit of analysis in the previous sections was the individual recordings. In this section we move one level up and place the focus at the country. We detect outlier countries in a similar manner as before where country features now summarise the information of the underlying recordings. The advantage of placing the focus at the country level is that the feature representations can now summarise the variety of styles that exist in the music of a country. Outliers are not judged by individual recordings but rather by the distribution of the whole set of recordings of each country.

We use *K*-means clustering to map recording representations to one of *K* clusters. The country representation is then derived from a histogram count of the *K* clusters of its recordings. Let $X\in R^{I\times J}$ denote the set of observations for *I* recordings and *J* features. We compute *K*-means for *X* and map recordings to one of *K* clusters. We use a linear encoding function $f:R^{J}\to R^{K}$ so that each recording representation $x_{i}\in R^{J}$ for *i* = 1, …, *I* is mapped to a vector ${\hat{x}}_{i}\in R^{K}$ via the dot product between **x**_*i*_ and the cluster centroids $m_{k}\in R^{J}$ for *k* = 1, …, *K* clusters. The feature vector for a country $c_{r}\in R^{K}$ is the normalised histogram count of *K* clusters for recordings *i* from country *r*, denoted

$$\begin{array}{c} c_{r}^{\prime }=\sum_{i}f\left(x_{i}\right). \end{array}$$ (6)

Each histogram is normalised to the unit norm, where $c_{r}=\frac{c_{r}^{\prime }}{∥c_{r}^{\prime }∥}$. Let $C\in R^{R\times K}$ denote the feature representations for *R* countries and *K* clusters derived as explained above. The optimal number *K* of clusters is decided based on the silhouette score [102] after evaluating *K*-means for *K* between 10 and 30 clusters.

We estimate similarity between countries via hierarchical clustering [103]. For consistency with the previous outlier detection method (section Outliers at the recording level), we use Mahalanobis distance to estimate pairwise similarity between countries. Pairwise Mahalanobis distance between countries is denoted

$$\begin{array}{c} D_{M}\left(c_{i},c_{j}\right)=\sqrt{{\left(c_{i}-c_{j}\right)}^{T}{\bar{S}}^{-1}\left(c_{i}-c_{j}\right)} \end{array}$$ (7)

where $\bar{S}$ is the covariance matrix and *i*, *j* ∈ {1, 2, …, *R*}. A hierarchy of countries is constructed using the average distance between sets of observations as the linkage criterion.

## Results

### Parameter optimisation

**Fig 6:**
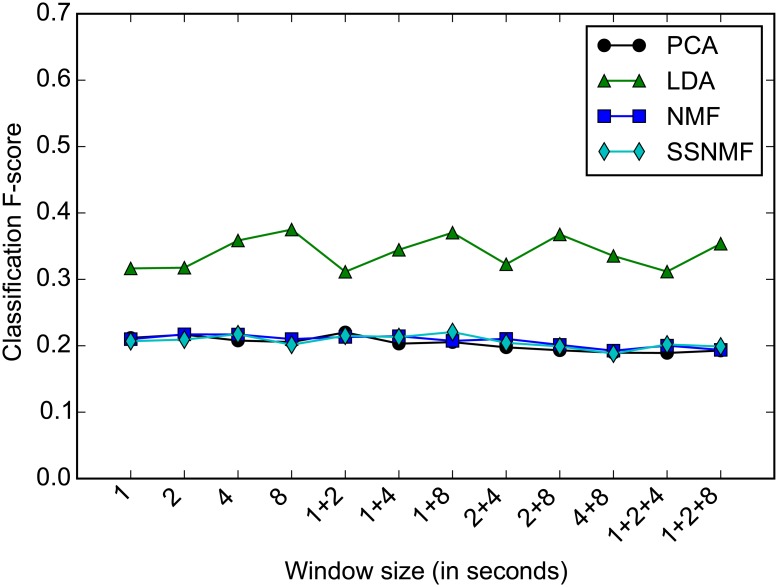
Classification F-score on the validation set for the best performing classifier (LDA) across different window sizes. Accuracies are compared for different feature learning methods (PCA, LDA, NMF, SSNMF). Combinations of window sizes are marked by ‘+’ in (a), for example ‘4+8’ represents the accuracy when combining features from the 4-second and the 8-second windows. Considering the performance of all feature learning methods, the optimal window size is 8 seconds.

As mentioned in the Audio content analysis section, the window size *w* in the feature extraction process (Features section) was optimised based on a classification task. Given the feature transformed representations of each recording in the training set, we trained 4 classifiers (KNN, LDA, SVM, RF), to predict the country label of a recording. Parameter optimisation was based on the classification accuracy on the validation data. We used the weighted average of the F-measure of each class [104], referred to as F-score, to report classification performance in this case of unbalanced data classes. Fig 6 shows the classification F-score of the best performing classifier (LDA) for a range of window sizes *w*. Based on this evaluation the optimal window size was *w* = 8 seconds with highest F-score of 0.37 for the LDA classifier in combination with the LDA-transformed features.

**Fig 7:**
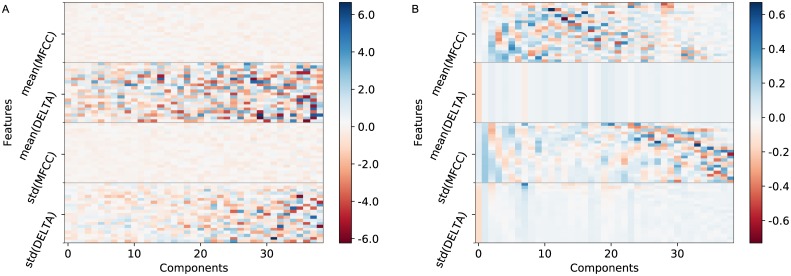
LDA and PCA components weigh timbral features in opposite ways. (A) LDA components focus on timbre fluctuation (mean and standard deviation of MFCC delta coefficients) over time. (B) PCA components focus on absolute timbre qualities (mean and standard deviation of MFCC coefficients) over time.

The dimensions of the LDA-transformed features can be explained in the following way. LDA components for the rhythmic features give more weight to the periodicities of the high-frequency Mel bands (above 1758 Hz). Melodic features receive similar weights for both the bases and activations of the pitch bi-histogram. LDA components for the harmonic features assign more weight to relative pitch values (mean of chroma vectors) rather than pitch fluctuations (standard deviation of chroma vectors) over time. LDA components for timbral features focus on timbre fluctuation (mean and standard deviation of MFCC delta coefficients) over time. This is opposite to the behaviour of PCA transformation where components focus on absolute timbre qualities (mean and standard deviation of MFCC coefficients) over time. Fig 7 illustrates the difference between LDA and PCA components for the timbral features.

### Classification

The classification results for the different classifiers in combination with the feature learning methods are presented in Table 1. Classification accuracy of the test set was assessed after fixing the window size of the feature extraction to *w* = 8 seconds as found optimal in section Parameter optimisation. Results suggest that the best classifier for our data when the combination of all features is considered is the LDA classifier with the LDA-transformed features (classification F-score of 0.321). Rhythmic, melodic, and harmonic features achieved best classification performance for the LDA-transformed features and the LDA classifier whereas timbral features achieved best classification performance for the LDA-transformed features and the SVM classifier. The first 10 components of the LDA classifier trained with the LDA-transformed features give more weight to the timbral and harmonic dimensions and explain 24% of the variance. The remaining components give more weight to the rhythmic and melodic dimensions. More information on the classification results and confusion matrices can be found in the published code repository (http://github.com/mpanteli/music-outliers).

**Table 1:** Classification F-scores of the test set for the country of recording (– denotes no transformation).

Transform	Classifier	F-score				
		All	Rhythm	Melody	Timbre	Harmony
LDA	LDA	0.321	0.150	0.070	0.199	0.107
SSNMF	LDA	0.183	0.053	0.039	0.165	0.082
NMF	LDA	0.178	0.059	0.046	0.166	0.086
–	LDA	0.177	0.060	0.038	0.191	0.084
PCA	LDA	0.175	0.055	0.046	0.162	0.084
LDA	KNN	0.152	0.055	0.023	0.282	0.086
SSNMF	KNN	0.143	0.043	0.015	0.227	0.072
PCA	KNN	0.141	0.053	0.027	0.221	0.081
–	KNN	0.140	0.052	0.027	0.222	0.082
NMF	KNN	0.114	0.043	0.029	0.178	0.080
–	RF	0.083	0.040	0.032	0.114	0.057
LDA	RF	0.071	0.031	0.017	0.150	0.051
NMF	RF	0.063	0.032	0.020	0.126	0.042
PCA	RF	0.046	0.026	0.019	0.140	0.045
SSNMF	RF	0.045	0.031	0.018	0.116	0.035
LDA	SVM	0.023	0.079	0.050	0.296	0.090
SSNMF	SVM	0.021	0.011	0.005	0.019	0.014
NMF	SVM	0.016	0.008	0.008	0.011	0.012
–	SVM	0.015	0.047	0.038	0.250	0.088
PCA	SVM	0.015	0.048	0.039	0.246	0.092

The window size of the features is 8 seconds as found optimal in section Parameter optimisation. Results are sorted by highest to lowest F-score of the combination of all features (‘All’).

### Outliers at the recording level

We found the optimal feature learning method (LDA) that best approximates music similarity in our data as defined by the classification task (Classification section). We use the LDA-projected space to investigate music dissimilarity and identify outliers in the dataset.

**Fig 8:**
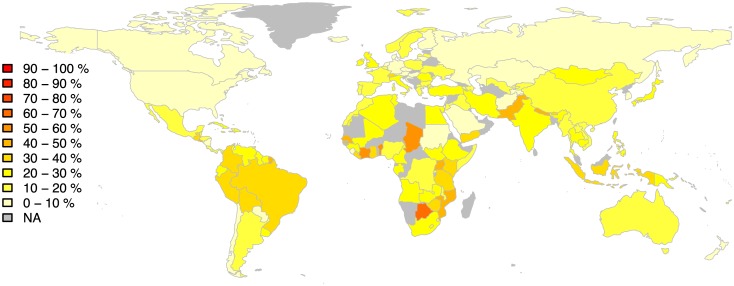
Distribution of outliers per country. The colour scale corresponds to the normalised number of outliers per country, where 0% indicates that none of the recordings of the country were identified as outliers and 100% indicates that all of the recordings of the country are outliers.

From a total number of 8200 recordings we identify 1706 recordings as outliers. The distribution of outliers per country, normalised by the number of recordings per country in our dataset, is summarised in Fig 8. We observe that the country with the most outliers is Botswana with 61% (55 out of 90) of its recordings identified as outliers, followed by Ivory Coast (60%, 9 out of 15), Chad (55%, 6 out of 11), and Benin (54%, 14 out of 26). The percentage of outliers per country was not significantly correlated with the number of recordings sampled from that country (Pearson correlation coefficient *r* = −0.01 with *p*-value = 0.91).

Listening to some examples we summarise the following timbral characteristics for the outliers. Outlier recordings from Botswana include solo performances of the mouthbow and dance songs featuring group singing accompanied with handclapping or other percussion. Outlier recordings from Ivory Coast feature music from the Kroo ethnic group who originated in eastern Liberia and consist of polyphonic music with singing accompanied by woodwind and guitar instruments. Outlier recordings from Chad feature mainly dance music with emphasis on percussive and wind instruments as well as examples of the singing voice in solo and group performances. Outliers from French Guiana feature solo flute performances and singing with percussive accompaniment. Outlier recordings from Gambia include examples of group singing with percussive accompaniment of drums, jingles and wooden blocks, solo performances of the gong and flute. Outlier recordings from Benin include solo performances of the Yoruba drums and music from the Fon culture including examples of group singing with gong accompaniment.

**Fig 9:**
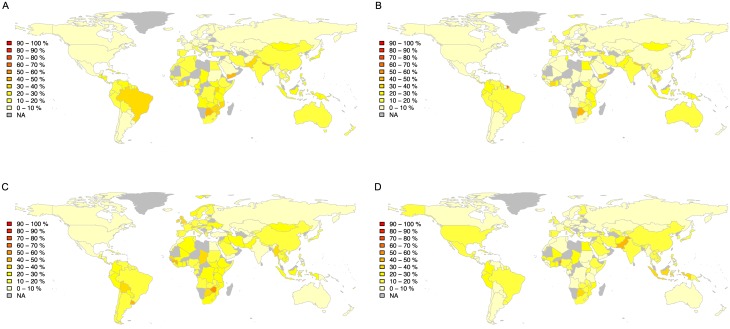
Distribution of outliers per country for each feature. Outliers detected for features of (A) rhythm, (B) timbre, (C) melody, and (D) harmony. The colour scale corresponds to the normalised number of outliers per country, from 0% of outliers (light colours) to 100% (dark colours).

To gain a deeper understanding of the type of outliers for each country we detect outliers using a) rhythmic, b) timbral, c) melodic, and d) harmonic features. Results are shown in Fig 9. With respect to rhythmic aspects the countries with the most outliers are Benin (50%, 13 out of 26), Botswana (49%, 44 out of 90), and Nepal (42%, 40 out of 95). The countries with the most outliers with respect to timbral characteristics are French Guiana (78%, 19 out of 28), Botswana (48%, 43 out of 90), and Ivory Coast (40%, 5 out of 13). The countries with the most outliers with respect to melodic aspects are Zimbabwe (53%, 8 out of 15), Uruguay (48%, 15 out of 31), and Guinea (46%, 5 out of 11) and with respect to harmonic aspects Benin (54%, 14 out of 26), Pakistan (46%, 42 out of 91), and Gambia (36%, 18 out of 50).

Listening to some examples we summarise the following characteristics for the outliers. Rhythmic outliers include examples from African polyrhythms as well as examples with frequent transitions between binary and ternary subdivisions. The most prominent instruments in the rhythmic outliers are pitched and non-pitched percussion. Most rhythmic outliers tend to have a ‘full’ rhythm, i.e. there are many onsets within each bar duration. Outliers with respect to timbral characteristics include solo performances of xylophones and gongs for example recordings from Botswana, Indonesia, and Gamelan recordings from the Philippines. Another category of instruments that often gives rise to timbre outliers are wind instruments such as reedpipes and flutes. Outliers with respect to melodic characteristics include polyphonic melodies performed on the accordion (e.g. recordings from Uruguay) or the mbira (e.g. recordings from Zimbabwe). With respect to harmony, outliers exhibit microtonal scales and feature instruments with distinct tuning, for example solo sitar or surnai performances from Pakistan, xylophone and gong performances from Benin and Indonesia. Listening examples can be found at the online demo (see http://mpanteli.github.io/music-outliers/demo/outliers).

#### Spatial outliers

**Fig 10:**
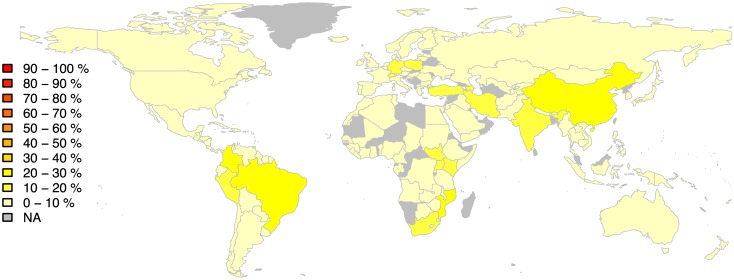
Distribution of outliers per country for the spatial neighbourhoods shown in S1 Table. The colour scale corresponds to the normalised number of outliers per country, from 0% of outliers (light colours) to 100% (dark colours).

In the previous section we detected outliers by comparing a recording to all other recordings in the dataset. Here we take into account spatial relations and we compare recordings from a given country only to recordings of its neighbouring countries (section Spatial neighbourhoods). We summarise the distribution of spatial outliers, normalised by the total number of recordings in each spatial neighbourhood, in Fig 10. Results show that China is the country with the most spatial outliers (26%, 26 out of 100), followed by Brazil (24%, 24 out of 100), Colombia (21%, 19 out of 90), and Mozambique (21%, 7 out of 34).

China is the country with most spatial neighbours in our dataset, bordering with 12 other countries for which we have music data (S1 Table). Recordings from China feature the butterfly harp string instrument and singing examples from the Han cultural group, often with a bright sound and prominent singing in relatively high frequencies. These examples are compared to various instruments and music styles from the neighbouring countries including lute performances from Kyrgyzstan, Mongolian jewish harp, Indian tala, Nepalese percussion and wind instrument performances, polyphonic singing from Vietnam and Laos, and instrumental pieces featuring the balalaika from Russia. Compared to the analysis of global outliers (Fig 8) we observe that recordings from China stand out only with respect to its spatial neighbourhoods but are not so distinct compared to the whole dataset of world music.

### Outliers at the country level

In this section we consider the country instead of the individual recordings as the unit of analysis and detect outlier countries as described in section Outlier countries.

**Fig 11:**
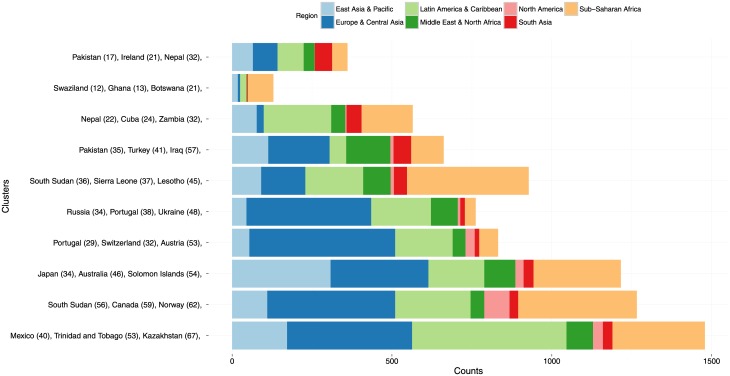
The top 3 countries for each of the 10 clusters.

The silhouette score indicated an optimal number of *K* = 10 clusters. We refer to the country labels of each recording to give an overview of the music styles captured in each cluster. The 3 most frequent countries in each cluster are shown in Fig 11.

**Fig 12:**
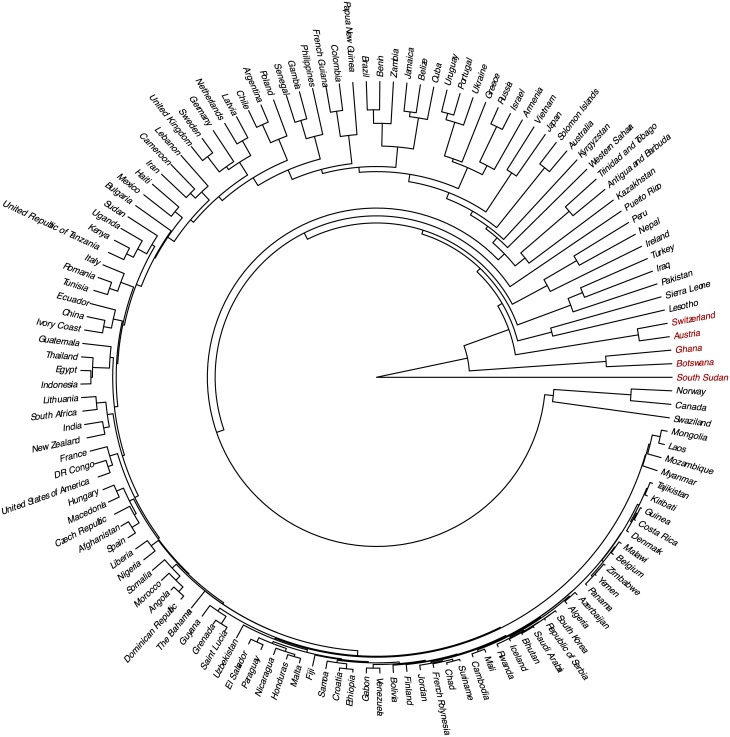
Hierarchical clustering of the 137 countries in our dataset. Each country was represented by the histogram of cluster mappings of its recordings (Outlier countries section). The most distinct countries are annotated with red colour.

The similarity between countries was estimated via hierarchical clustering. Results are presented in a dendrogram in Fig 12. The countries with the most distinct feature representations are South Sudan, Botswana, Ghana, Austria and Switzerland (in order of most to least distinct). The aforementioned countries were found dissimilar (with respect to a threshold) to any other country in our dataset.

Recordings from South Sudan feature mostly examples of the singing voice in solo and group performances. The use of solely the singing voice is what we believe makes the feature representation of South Sudan so different from other countries. A similar observation holds for recordings from Austria and Switzerland featuring mostly dance songs with accordion accompaniment. This might not be a unique music style across our dataset but the consistent use of this style in the recordings from Austria and Switzerland is what we think makes them most distinct from other countries. Botswana and Ghana, also detected as outlier countries with the hierarchical clustering approach, exhibit the use of a variety of music styles. Botswana was also detected as the country with the most outlier recordings compared to the global dataset (section Outliers at the recording level). We note that Fig 12 also revealed some music similarity relationships between countries of geographical or cultural proximity. However, as the scope of this study is rather on music dissimilarity and outliers we leave the exploration of these relationships for future work.

## Discussion

We combined world music recordings from two large archives and proposed a methodology to extract music features and detect outliers in the dataset. We developed signal processing methods to process music information from the audio signal taking into account the challenges imposed by noisy and musically diverse recordings. Our analyses explored differences and similarities of world music and revealed geographical patterns of music outliers.

We took into account several pre-processing steps to isolate relevant music information from the audio signal: speech segments were separated from music, frequencies above 8000 Hz were omitted for consistency with old recording equipment, and low-level music descriptors were combined with feature learning to give higher-level representations robust to diverse music characteristics. The size of the texture window was optimised and we found that longer windows (8 seconds) provide better representations for our music data than shorter ones (4,2,1 seconds). Feature learning was better in the supervised setting (LDA outperformed PCA and NMF) even though class labels (in this case countries) were not necessarily unique identifiers for the underlying musical content.

We proposed a method to detect outliers and explored several ways of understanding the musical differences. We listed the countries with the most outlier recordings and expanded the analysis to explain which music features are distinct in these outlier recordings. For example, Botswana was the country with most of its recordings detected as outliers and feature analysis showed that those outliers were mostly due to rhythmic and timbral features. With respect to rhythmic features, African countries indicated the largest amount of outliers with recordings often featuring the use of polyrhythms. Harmonic outliers originated mostly from Southeast Asian countries such as Pakistan and Indonesia, and African countries such as Benin and Gambia with recordings often featuring inharmonic instruments such as the gong and bell.

We ran a sensitivity experiment to check how stable our outlier findings are with respect to different datasets. We repeated the outlier analysis 10 times, each time selecting at random a stratified sample of 80% of the original dataset. The majority vote of outlier countries resulting in the top *K* = 10 positions of each experiment was used as the ground truth. Assessing the precision at *K* = 10 for each experiment assuming majority vote ground truth showed that the geographical patterns of outliers (Fig 8) were on average consistent across multiple random subsets of the original dataset (precision at *K* mean = 0.67, standard deviation = 0.06).

Incorporating spatial information we were able to compare recordings from neighbouring countries. This gave rise to music cultures that are not distinct compared to the global dataset but are still unique compared to their spatial neighbours. For example, music from China with bright timbres was found to be unique compared to its many spatial neighbours. Music from Brazil was also distinct compared to its spatial neighbours, an observation that could be attributed to cultural differences such as the use of different languages between Brazil and its neighbouring countries. Proving historical and cultural influence is not the aim of this study but we believe our findings could provide a good starting point for further investigation.

We also proposed a method to extract feature summaries for each country and estimated clusters for the whole set of recordings. We found 10 clusters to best represent the music styles in our dataset and observed recordings from geographically similar regions often clustered together. Hierarchical clustering at the country level representation revealed African countries such as South Sudan, Botswana, and Ghana as most distinct from others in the dataset.

### Hubness

This research deals with high dimensional vectors and analysis of nearest neighbour relationships. High dimensional spaces are prone to produce data points that appear in the neighbourhood of other points disproportionately often. We tested the effect of hubness in our data following the approach suggested by Schnitzer et al. [105]. We measured hubness as the skewness of the *n*-occurrence where *n*-occurrence defines the number of times track *x* occurs in the top *n* neighbours of other tracks. We used pairwise Mahalanobis distances and assessed the *n* nearest neighbours for each track in our dataset for *n* = 60, the average number of recordings per country. We observed a positively skewed distribution with hubness = 10.1. A total of 129 out of 8200 recordings occurred in the nearest neighbour lists of more than 1000 tracks (2% large hubs) and 3332 recordings had *n*-occurrence = 0 (41% orphans). Pairwise Mahalanobis distances in this study are only used for the computation of outlier countries (section Outlier countries). Future work could aim to reduce hubness via local scaling or mutual proximity [105].

### Future work

There are several steps in the overall methodology that could be implemented differently and audio excerpts and features could be expanded and improved. Numerous audio features have been proposed in the literature for describing musical content in sound recordings for various applications. We selected a small set of features from the MIR domain based on their state-of-the-art performance and relevance for world music analysis. It is clear that any such set of features does not capture all aspects of a set of musical recordings. Future work could explore the suitability of feature sets proposed by ethnomusicologists [20] or embeddings learned from raw audio or spectrograms [106].

We used linear feature learning methods to learn higher-level representations from our low-level descriptors. Depending on the data and application, more powerful non-linear methods could be employed to learn meaningful feature representations [107]. What is more, our analysis relies on a bag-of-frames approach where temporal information of the entire music piece is lost by averaging short frames across time. Although this approach is in line with state of the art MIR research [87, 90] alternative methods capturing temporal relationships such as Hidden Markov Models [108] could be considered.

Like all studies of this nature our study is subject to sampling bias. Our observations on world music similarity are restricted to the dataset we analyse. It is difficult to gather representative samples of ‘all’ music of the world. We aimed to maximise geographical spread in the dataset by including as many countries as possible and representative samples from each country were drawn at random. This resulted in a total of 137 countries with a minimum of 10 recordings per country. Even though this is the largest and most diverse corpus of world music studied so far, there are many areas of the world and cultures that are not represented. The creation of a representative world music corpus will continue indefinitely as more music is recorded and the digitisation of archived recordings proceeds.

In this study country labels have been considered a proxy to music style and have been used to train models for music similarity and dissimilarity. While countries provide a broad notion of ethnic boundaries, music styles are not homogeneous within these boundaries. A country may exhibit several music styles and a music style may spread across many countries. The ambiguity of these boundaries provides an upper limit to the performance of our models. This ambiguity could be reduced by incorporating more information, for example the culture or language of the musicians, to better approximate the music style of a recording. Extracting culture or language information from the currently available metadata requires additional manual labour and this is a task left for future work.

Furthermore, a lot of information regarding the music style of a recording can be extracted from the date it was created. Music evolves over time, and two recordings from the same location but recorded with a time difference of 50 years may vary in their style. In this study we ignored temporal information and considered our dataset as a static collection of world music. Country of origin and recording date could be used together to define the music style of a recording.

Our study focuses on the detection of outliers in music collections. The data we work with are numerical representations derived from a multi-step procedure of processing the audio signal. The suitability of the audio tools can be questioned with regard to their ability to capture and represent high-level musical concepts [70]. Likewise, the patterns we observe can sometimes be artifacts of the tools we use. We note that in this study the estimated outliers did not appear to be attributable to recording date differences or acoustic environments but quantitative and qualitative evaluation could be expanded [109].

## Conclusion

The comparison of world music cultures has been traditionally studied with non-computational tools. We investigated similarity in a large corpus of world music using signal processing and data mining tools. We analysed thousands of recordings from folk and traditional music from around the world and quantified differences and similarities. Our findings identify regions that have possibly developed unique musical characteristics such as Botswana, as well as China, which is most distinct from its neighbours. We have also explored geographical patterns of music outliers for different sets of features and found that Benin has the most outlier recordings with respect to rhythm and harmony, French Guiana with respect to timbre, and Zimbabwe with respect to melody. A categorisation into world music styles identified 10 clusters with South Sudan and Botswana exhibiting the most distinct use of these clusters. This is the first study to consider the computational analysis of such a large world music corpus. There is a lot to be explored yet and we believe continuing on this line of research will help us understand better the music cultures of the world.

## Supporting information

S1 Table Spatial neighbours for each country in our dataset. (PDF)
