# Supplementary material for: A computational study on outliers in world music
Source: PLoS One. 2017 Dec 18;12(12):e0189399. doi: 10.1371/journal.pone.0189399 (PMC5734747; doi:10.1371/journal.pone.0189399)
Supplement: S1 Table — (PDF) [file pone.0189399.s001.pdf]

Country	Spatial neighbours
Afghanistan	Tajikistan, Pakistan, Uzbekistan, China, Iran
Algeria	Morocco, Tunisia, Mali, Western Sahara
Angola	Zambia, DR Congo
Antigua and Barbuda	Saint Lucia
Argentina	Uruguay, Chile, Bolivia, Paraguay, Brazil
Armenia	Azerbaijan, Turkey, Iran
Australia	Fiji, Papua New Guinea, Indonesia
Austria	Hungary, Germany, Czech Republic, Switzerland, Italy
Azerbaijan	Turkey, Russia, Armenia, Iran
Belgium	Germany, France, Netherlands
Belize	Guatemala, Mexico
Benin	Nigeria
Bhutan	India, China
Bolivia	Peru, Argentina, Chile, Paraguay, Brazil
Botswana	Zambia, Zimbabwe, South Africa
Brazil	Uruguay, Argentina, French Guiana, Bolivia, Guyana, Suriname, Colombia, Venezuela, Paraguay, Peru
Bulgaria	Romania, Macedonia, Greece, Turkey
Cambodia	Thailand, Laos, Vietnam
Cameroon	Gabon, Nigeria, Chad
Canada	United States of America
Chad	Nigeria, South Sudan, Cameroon
Chile	Argentina, Bolivia, Peru
China	Afghanistan, Kazakhstan, Kyrgyzstan, Laos, Russia, Bhutan, Mongolia, Myanmar, India, Tajikistan, Pakistan, Vietnam
Colombia	Panama, Ecuador, Venezuela, Peru, Brazil
Costa Rica	Panama, Nicaragua
Croatia	Hungary
Cuba	The Bahamas, Jamaica, Haiti
Czech Republic	Poland, Germany, Austria
DR Congo	United Republic of Tanzania, Angola, Rwanda, Zambia, South Sudan, Uganda
Denmark	Germany
Dominican Republic	Haiti
Ecuador	Peru, Colombia
Egypt	Israel, Sudan
El Salvador	Honduras, Guatemala
Ethiopia	South Sudan, Kenya, Sudan, Somalia
Fiji	Papua New Guinea, Solomon Islands, New Zealand
Finland	Norway, Sweden, Russia
France	Belgium, Germany, Italy, Switzerland, Spain
French Guiana	Suriname, Brazil
French Polynesia	Samoa, Mexico
Gabon	Cameroon
Gambia	Senegal
Germany	Poland, France, Austria, Belgium, Netherlands, Switzerland, Czech Republic, Denmark
Ghana	Ivory Coast
Greece	Bulgaria, Macedonia, Turkey

Grenada	Trinidad and Tobago, Antigua and Barbuda, Saint Lucia
Guatemala	El Salvador, Belize, Honduras, Mexico
Guinea	Liberia, Senegal, Sierra Leone, Ivory Coast, Mali
Guyana	Suriname, Venezuela, Brazil
Haiti	Dominican Republic
Honduras	El Salvador, Guatemala, Nicaragua
Hungary	Croatia, Romania, Ukraine, Austria
Iceland	Ireland, Netherlands, United Kingdom
India	Afghanistan, Bhutan, Myanmar, Nepal, China, Pakistan
Indonesia	Papua New Guinea, Malaysia
Iran	Afghanistan, Armenia, Azerbaijan, Iraq, Pakistan, Turkey
Iraq	Saudi Arabia, Jordan, Turkey, Iran
Ireland	United Kingdom
Israel	Egypt, Lebanon, Jordan
Italy	France, Switzerland, Austria
Ivory Coast	Liberia, Ghana, Mali, Guinea
Jamaica	Haiti, Cuba, The Bahamas
Japan	Philippines, South Korea
Jordan	Iraq, Saudi Arabia, Israel
Kazakhstan	Kyrgyzstan, Uzbekistan, Russia, China
Kenya	South Sudan, United Republic of Tanzania, Ethiopia, Somalia,Uganda
Kiribati	Guyana, Suriname, Brazil
Kyrgyzstan	Kazakhstan, Uzbekistan, China, Tajikistan
Laos	Thailand, Cambodia, Myanmar, Vietnam, China
Latvia	Lithuania, Russia
Lebanon	Israel
Lesotho	South Africa
Liberia	Sierra Leone, Ivory Coast, Guinea
Lithuania	Poland, Latvia, Russia
Macedonia	Bulgaria, Greece
Malawi	United Republic of Tanzania, Zambia, Mozambique
Malaysia	Thailand, Indonesia
Mali	Algeria, Senegal, Ivory Coast, Guinea
Malta	Italy, Tunisia, Greece
Mexico	United States of America, Belize, Guatemala
Mongolia	Russia, China
Morocco	Algeria, Western Sahara, Spain
Mozambique	United Republic of Tanzania, Zambia, Zimbabwe, Malawi, South Africa, Swaziland
Myanmar	Thailand, Laos, India, China
Nepal	India, China
Netherlands	Belgium, Germany
New Zealand	Fiji, Solomon Islands, Australia
Nicaragua	Costa Rica, Honduras
Nigeria	Cameroon, Benin, Chad
Norway	Finland, Sweden, Russia
Pakistan	Afghanistan, India, China, Iran
Panama	Costa Rica, Colombia
Papua New Guinea	Indonesia
Paraguay	Argentina, Bolivia, Brazil

Peru	Ecuador, Colombia, Chile, Bolivia, Brazil
Philippines	Malaysia, Vietnam, Indonesia
Poland	Lithuania, Germany, Czech Republic, Russia, Ukraine
Portugal	Spain
Puerto Rico	Antigua and Barbuda, Dominican Republic
Republic of Serbia	Croatia, Macedonia, Hungary
Romania	Bulgaria, Hungary, Ukraine
Russia	Kazakhstan, Poland, Finland, Latvia, Azerbaijan, Lithuania, Mongolia, China, Norway, Ukraine
Rwanda	United Republic of Tanzania, DR Congo, Uganda
Saint Lucia	Trinidad and Tobago, Antigua and Barbuda, Grenada
Samoa	Mexico, French Polynesia
Saudi Arabia	Iraq, Yemen, Jordan
Senegal	Gambia, Mali, Guinea
Sierra Leone	Liberia, Guinea
Solomon Islands	Fiji, Papua New Guinea
Somalia	Kenya, Ethiopia
South Africa	Zimbabwe, Mozambique, Swaziland, Botswana, Lesotho
South Korea	China, Japan, Philippines
South Sudan	Kenya, Ethiopia, DR Congo, Sudan, Uganda
Spain	Portugal, France, Morocco
Sudan	Egypt, South Sudan, Ethiopia, Chad
Suriname	Guyana, French Guiana, Brazil
Swaziland	Mozambique, South Africa
Sweden	Norway, Finland
Switzerland	Germany, Italy, France, Austria
Tajikistan	Afghanistan, Kyrgyzstan, Uzbekistan, China
Thailand	Malaysia, Cambodia, Myanmar, Laos
The Bahamas	Haiti, Cuba, Jamaica
Trinidad and Tobago	Venezuela, Grenada, Saint Lucia
Tunisia	Algeria
Turkey	Armenia, Azerbaijan, Greece, Bulgaria, Iran, Iraq
Uganda	South Sudan, Kenya, United Republic of Tanzania, DR Congo, Rwanda
Ukraine	Poland, Hungary, Romania, Russia
United Kingdom	Ireland
United Republic of Tanzania	Kenya, Rwanda, Zambia, Malawi, Mozambique, DR Congo, Uganda
United States of America	Canada, Mexico
Uruguay	Argentina, Brazil
Uzbekistan	Afghanistan, Kazakhstan, Kyrgyzstan, Tajikistan
Venezuela	Guyana, Colombia, Brazil
Vietnam	Cambodia, Laos, China
Western Sahara	Algeria, Morocco
Yemen	Saudi Arabia
Zambia	United Republic of Tanzania, Angola, Zimbabwe, Malawi, Botswana, Mozambique, DR Congo
Zimbabwe	Zambia, Mozambique, South Africa, Botswana
